# Prototyping a System for Truck Differential Lock Control

**DOI:** 10.3390/s19163619

**Published:** 2019-08-20

**Authors:** Pavel Kučera, Václav Píštěk

**Affiliations:** Institute of Automotive Engineering, Brno University of Technology, Technická 2896/2, 616 69 Brno, Czech Republic

**Keywords:** sensors, mechatronic system, truck, differential lock, prototyping, language C, NI VeriStand

## Abstract

The article deals with the development of a mechatronic system for locking vehicle differentials. An important benefit of this system is that it prevents the jamming of the vehicle in difficult adhesion conditions. The system recognizes such a situation much sooner than the driver and is able to respond immediately, ensuring smooth driving in off-road or snowy conditions. This article describes the control algorithm of this mechatronic system, which is designed for firefighting, military, or civilian vehicles with a drivetrain configuration of up to 10 × 10, and also explains the input signal processing and the control of actuators. The main part of this article concerns prototype testing on a vehicle. The results are an evaluation of one of the many experiments and monitor the proper function of the developed mechatronic system.

## 1. Introduction

There is an increasing trend toward introducing mechatronic systems wherever possible. It is, therefore, no wonder that a certain type of mechatronic system is an integral part of every manufactured car. To speed up the development of such systems, new methods and various sophisticated tools are constantly being designed, with the aim to reduce the time and cost of development. Many intelligent mechatronic systems [1,2,3,4,5,6] being developed are related to the chassis and powertrain of vehicles. This article also deals with the development of a system associated with the powertrain of vehicles or, more specifically, the development of a mechatronic system for an automatic differential lock.

The basic function of this system is to evaluate the slip of the wheels and powertrain shafts. The control system evaluates sensor signals from wheel speed sensors, the vehicle pedals, an air pressure sensor in the pneumatic circuit, feedback sensors, control switches and buttons, Controller Area Network—CAN messages from other Electronic Control Units—ECUs, and touch displays. The system then sends the signals to the actuators which are assembled from the electrovalve, the pneumatic circuit, a feedback sensor, and a special dog clutch. When the electrovalve is opened, pressurized air is introduced into the pneumatic cylinder, thereby moving its piston with the bracket; this locks the special dog clutch. These actuators are located in the appropriate differentials or used to connect the front axle input shafts to the transfer case of the vehicle to activate all-wheel drive. The driver controls the system with three switches and one button. The first switch is used to set the automatic and manual control modes. The two other switches and the button are used to activate all-wheel drive and lock the rear inter-differentials, the rear axle-differentials, and the front axle differentials in manual control mode. Another option is to set up three driving modes for road, field, and terrain/snow on the touch display. Information on all-wheel drive activation or locking the relevant differentials is also provided on the display.

This system was developed to improve the properties of the vehicle’s powertrain, improve fuel economy, and reduce tire wear. A vehicle fitted with this system is more environmentally friendly and protects the powertrain against inappropriate differential lock control by inexperienced drivers, therefore, the system is controlled automatically. The testing and the evaluation of this system was carried out in the form of prototyping, where a controller with a control algorithm was connected to the vehicle prototype. The powertrain of the vehicle prototype consisted of an engine, a transmission, a transfer case, a rear inter-differential, and four axles with an axle-differential. The powertrain enabled the front axles drive to be activated.

In the area of drive control, differential lock, and all-wheel drive activation, the Zahnradfabrik Friedrichshafen Automatic Drive-Train Management—ZF ADM differential locking system described in [7,8] can be used for trucks. Another system is the Meritor driver-controlled differential lock (DCDL) (see [9]). These two companies created a new ZF Meritor, so it can be assumed that DCDL is the same system as ZF ADM, i.e., a system that evaluates wheel slip. The control algorithm evaluates slip and locks or unlocks the relevant differentials. A dog clutch is used in the differential. There are a number of systems on the market that control torque distribution and the locking of differentials in passenger cars, such as Torque Vectoring. However, these systems cannot currently be used for trucks or special vehicles due to their high transmission torque. For this reason, it is necessary to use a dog clutch for differential locking. A similar system is introduced in this article describing the principle of the developed control algorithm and prototype testing on a vehicle.

## 2. The Vehicle Prototype

**Figure 1:**
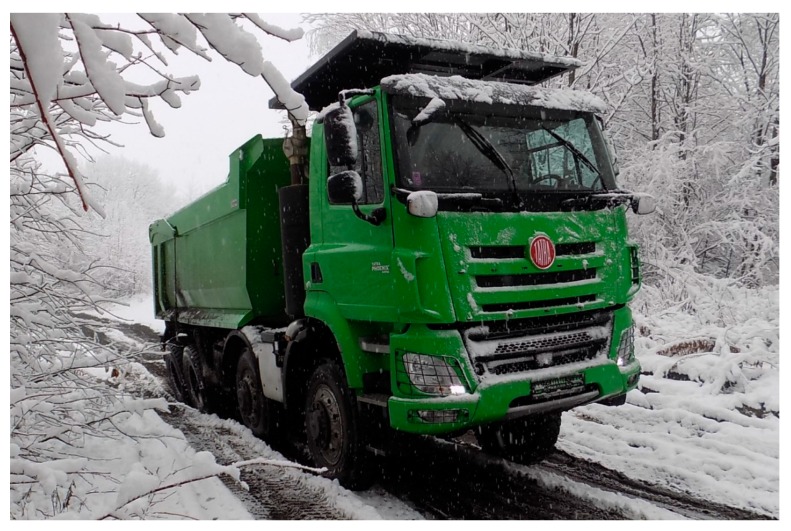
Prototype of a civilian vehicle.

In order to carry out the testing, it was necessary to produce a vehicle prototype with the mechanical parts corresponding to the differential lock system. The prototype vehicle is shown in Figure 1. The vehicle was developed by the client and is used by civilians. 

The prototype was based on the modification and development of new mechanical parts for the serial vehicle powertrain. This involved the development of special dog clutches designed for the quick locking/unlocking of differentials when the vehicle is being driven. Emphasis was placed on resistance to shock torque. This development of the dog clutch and parts of the powertrain took place in cooperation with the vehicle manufacturer. Computer Numeric Control—CNC technology [10,11,12,13] and grinding were used for the production of the dog clutches. The dog clutch variants were tested in detail for functionality and durability on a test bench prior to installation in a test vehicle. The vehicle prototype was largely constructed from modified mechanical components. The dog clutches were located in each differential and were also used to connect the front axle drive shafts. The 8 × 8 drive prototype was assembled from 4 axle differentials, one inter-axle differential for the rear axles, and two dog clutches to activate the drive of the front axles. All of these dog clutches are forced by the pneumatic cylinder with a bracket, with an electrovalve connected to it. When an electrovalve is opened, it releases compressed air into the pneumatic circuit. 

**Figure 2:**
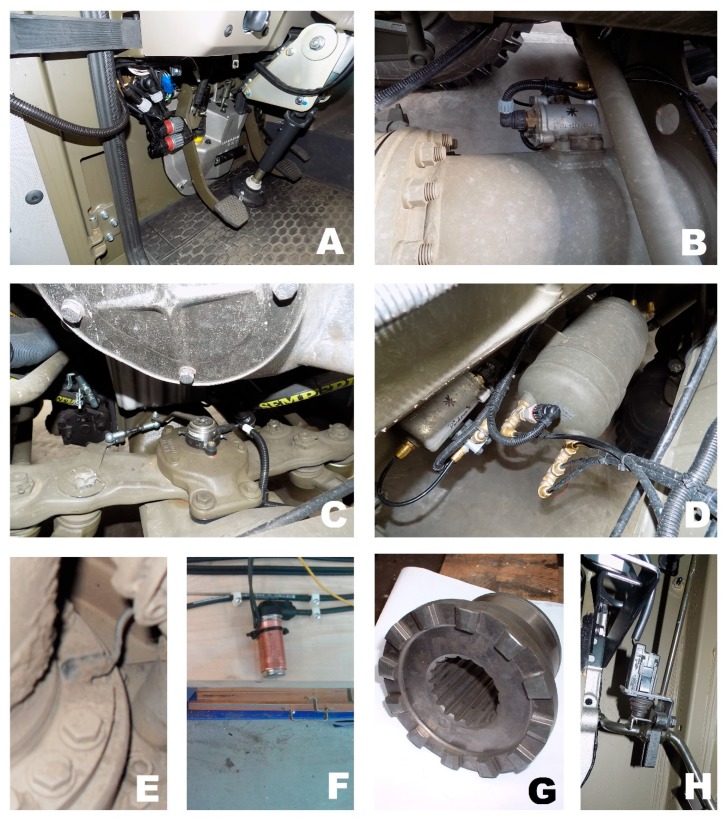
Sensors and actuators. (**A**) Figure caption; (**B**) figure caption; (**C**) figure caption; (**D**) figure caption; (**E**) figure caption; (**F**) figure caption; (**G**) figure caption; (**H**) figure caption.

The compressed air forces a displacement of the movable part of the dog clutch and locks it. The dog clutch is unlocked when the electrovalve closes and compressed air starts to leave the pneumatic cylinder due to the piston being pressed by an inner spring. The pneumatic cylinder includes a feedback switch sensor that signals the locking of the dog clutches. This signals the locking of the differentials or the activation of the front axle drive. This assembly of mechanical elements, i.e., an electrovalve, a pneumatic element, and a sensor, is an actuator, which is monitored and controlled by a developed algorithm. The illustration of the pneumatic cylinder and the electovalve are shown in Figure 2B. A picture of a dog clutch is shown in Figure 2G. In addition, the prototype was fitted with speed sensors (Figure 2E,F) that are commonly used in Anti-lock Brake System—ABS. In the case of the axles with the ABS speed sensors, it was from these wheels that the CAN messages with wheel speed information were received. The vehicle also had a steering wheel sensor, which is shown in Figure 2C. Figure 2D denotes a picture of the pneumatic circuit air reservoir, which is supplemented by a pressure sensor. An older prototype of the vehicle did not have any CAN messages with the value of the pedals. Therefore, extra pedal sensors were used, as shown in Figure 2A indicating the clutch/brake pedal; Figure 2H shows the accelerator pedal. For the newer prototype of the vehicle, these additional sensors were not needed because they were used normally. Their values are available in basic CAN messages.

## 3. Control Algorithm

**Figure 3:**
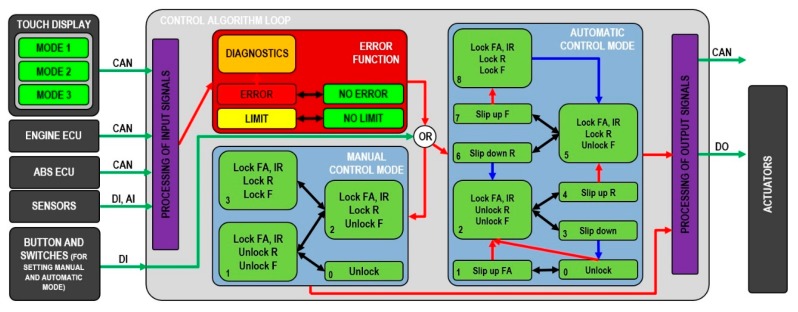
Diagram of the control algorithm (DI—Digital inputs, AI—Analog inputs, DO—Digital outputs).

This section describes the algorithm that was developed for the automatic locking of differentials for trucks, special vehicles, and tractors. The aim of this development was to create a system that would make it easier to drive on difficult terrain. This would help the driver to take the vehicle into extremely inaccessible places without getting stuck. Therefore, it was necessary to create the code of a control algorithm, the diagram of which is shown in Figure 3.

### 3.1. Main Loop of the Control Algorithm

Sensor inputs for the control algorithm are processed by code in the main loop (in the National Instruments—NI 3110 hardware) or in the Field Programmable Gate Array—FPGA (NI 9159) for fast processing. In the main loop, the control algorithm decides the system response to the current situation. This loop contains sub functions with a period of 10 ms. This loop is a summary of the main functions of the control algorithm and it decides whether all-wheel drive will be activated or the relevant differentials will be locked. The control algorithm inputs include wheel speeds, steering angle, the shaft speed difference of a locked dog clutch, vehicle velocity, actuator feedback, pedal signals, vehicle braking status, pneumatic circuit pressure, engine torque, switches and button signals, and driving mode setting. The control algorithm inputs are evaluated from sensors, CAN messages from other ECU, control switches and the button, and touch display information. These are shown in Figure 3.

The basic function of the system is the evaluation of the slip of the wheels and shafts. Therefore, the following equation is used to evaluate the slip:

(1) $$slip_{i}=\frac{\max\left(omg_{j},omg_{k}\right)-\min\left(omg_{j},omg_{k}\right)}{\max\left(omg_{j},omg_{k}\right)}-slipC_{i},$$

where *omg* is the speed of the respective wheels or shafts, *i* is the index of the respective slip or the correction slip, *j* and *k* are the wheel or shaft indexes, and *slipC* is the correction slip. If the function max is equal to 0, it is treated to prevent division by zero. The resulting slip is compared to the slip control parameter. If the slip value is higher than the control parameter, the control algorithm responds to this situation. For the slip evaluation, it is necessary to monitor the wheel speed values. Wheel speed values are monitored using two approaches: From the CAN message (Parameter Group Number: 0xFEBF), the wheel speed (km/h) information is read. These messages are sent by an ABS system, but only for four wheels. This means that the values from the CAN message have to be converted to wheel speed *omg* (rpm). Therefore, wheel circumference is defined and is recalculated to wheel radius for the subsequent processing of wheel speed. The dependence of the wheel radius on the load or its wear is included in the control algorithm, so the slip control parameter must be set with sufficient margin. This means that if a slip is evaluated due to the different radii of the wheels, there is no locking of the differentials or activation of all-wheel drive. Other wheels are equipped with other speed sensors. Their signal is monitored and evaluated in the FPGA code. Both approaches obtain the speed: *omgFLI*—the left wheel speed of the first front axle of the vehicle; *omgFRI*—the right wheel speed of the first front axle of the vehicle; *omgFLII*—the left wheel speed of the second front axle of the vehicle; *omgFRII*—the right wheel speed of the second front axle of the vehicle; *omgRLI*—the left wheel speed of the first rear axle of the vehicle; *omgRRI*—the right wheel speed of the first rear axle of the vehicle; *omgRLII*—the left wheel speed of the second rear axle of the vehicle; and *omgRRII*—the right wheel speed of the second rear axle of the vehicle. The speed of the drive shafts are also calculated from these wheel speeds. The following is the use of Equation (1) for the slip calculation, where the results are: *sFLRI*—the slip between the right and left wheels of the first front axle of the vehicle; *sFLRII*—the slip between the right and left wheels of the second front axle of the vehicle; *sRLRI*—the slip between the right and left wheels of the first rear axle of the vehicle; *sRLRII*—the slip between the right and left wheels of the second rear axle of the vehicle; *sAIR*—the slip between the output shafts of the rear inter-differential; *sAFRI*—the slip between the input shaft of the first front axle and the output shaft of the transfer case; and *sAFRII*—the slip between the input shaft of the second front axle and the output shaft of the transfer case.

The value of the steering angle is used for the evaluation of the correction slip and is applied in Equation (1). The principle is to evaluate the relative slip caused by turning the vehicle. For this purpose, Ackermann steering geometry is used. The value of the steering angle is also used for axle-differential lock limitation, where axle-differentials are unlocked when the steering angle limit is exceeded. 

The shaft speed difference of a locked dog clutch is evaluated by the difference of the respective shaft speeds. These include: *doFLRI*—the speed difference between the output shafts of the axle-differential of the first front axle of the vehicle; *doFLRII*—the speed difference between the output shafts of the axle-differential of the second front axle of the vehicle; *doRLRI*—the speed difference between the output shafts of the axle-differential of the first rear axle of the vehicle; *doRLRII*—the speed difference between the output shafts of the axle-differential of the second rear axle of the vehicle; *doAIR*—the speed difference between the output shafts of the rear inter-differential; *doAFRI*—the speed difference between the input shaft of the first front axle and the output shaft of the transfer case; and *doAFRII*—the slip between the input shaft of the second front axle and the output shaft of the transfer case.

Vehicle speed *vc* is calculated from the minimum speed of all wheels when the vehicle is not braking. When the vehicle is braking, the vehicle speed is calculated from the maximum speed of all wheels.

In addition, the feedback switch value is monitored. This sensor is placed in all of the pneumatic cylinders, which are parts of the actuators. The sensor signals the actual activation of all-wheel drive or the locking of the relevant differentials. These include: *FeedbackFAI*—the input value of the algorithm sent by the actuator to get feedback on the activation of the first front axle drive; *FeedbackFAII*—the input value of the algorithm sent by the actuator to get feedback on the activation of the second front axle drive; *FeedbackIR*—the input value of the algorithm sent by the actuator to get feedback on the locking/unlocking of the rear inter-differential; *FeedbackRI*—the input value of the algorithm sent by the actuator to get feedback on the locking/unlocking of the axle-differential of the first rear axle of the vehicle; *FeedbackRII*—the input value of the algorithm sent by the actuator to get feedback on the locking/unlocking of the axle-differential of the second rear axle of the vehicle; *FeedbackFI*—the input value of the algorithm sent by the actuator to get feedback on the locking/unlocking of the axle-differential of the first front axle of the vehicle; and *FeedbackFII*—the input value of the algorithm sent by the actuator to get feedback on the locking/unlocking of the axle-differential of the second front axle of the vehicle.

Pedal signals are read from sensors or from CAN messages. These include: *sigB*—brake pedal signal; *sigT*—accelerator pedal signal; and *sigC*—clutch pedal signal. The variable *sigB* also includes the braking signals from the engine, the parking brake, and the retarder. If the value *sigB* is 1, the algorithm does not enable the activation of any actuator to maintain the stability of the vehicle. 

The pressure value *sigP* is monitored for the evaluation of air pressure in the system circuit. This prevents damage to the mechatronic system if the pressure is out of range.

From the engine ECU, a CAN message is transmitted with the current engine torque value. Therefore, this value is processed and also used as the input value of the control algorithm. If the engine torque value is large, the control algorithm does not allow the locking of the relevant dog clutch in the actuators.

The driver uses three switches and one button to set the control algorithm. The first switch is used to set the automatic and manual control modes of the algorithm. The other two switches and the button are used to activate all-wheel drive and lock the rear inter-differentials, the rear axle-differentials, and the front axle-differentials in manual control mode.

Another option the driver has is to set up three control modes on the touch display for road, field and terrain/snow. Mode 1 is for road, mode 2 is for field roads, and mode 3 is for terrain/snow. The functions for setting the control parameters according to the selected user system modes in the algorithm are as follows.

Before the automatic or manual control mode function is running, the presence of system errors must be checked by using the error function, which will be described in more detail in the next section. The algorithm evaluates the response to the vehicle’s current behavior. Within the development, two approaches to the control algorithm were tested. One was partially independent and is described in [14] and the other was dependent differential locking and is described here. This process is shown in Figure 3. In this case, all-wheel drive and the locking of the inter-differentials are activated in the first phase (level 2/1). In the next phase, all rear axial differentials are locked (level 5/2), and all front axial differentials are locked in the final third phase (level 8/3). When the control algorithm loop is started, it is first decided whether there is a fault in the system. If there is a fault in the system, depending on its priority, the control algorithm is set to one of three options. The first state is that the control algorithm works in both manual and automatic mode. The second state occurs when the fault is more severe and the automatic control mode is significantly dependent on it. In this case, the system is switched to manual control mode regardless of the control mode set by the user. If the errors are considered very dangerous, the system is deactivated. If the system has no serious failure, it is controlled by the set value of the automatic or manual control switch set by the user. 

In the case of manual control mode, differential locking occurs according to the left side of the diagram shown in Figure 3. The diagram is divided into 4 levels. If the driver wants to activate all-wheel drive or lock the differential, the procedure is as follows. In the first stage, the driver presses the first switch on the dashboard and the algorithm changes the value *LockFAI-II* and *LockIR* to 1 (level 1) if the other checked variables of the engine torque and the speed difference between the shafts are below the limit. In the second stage, the driver presses the second switch and the algorithm changes the value *LockRI-II* to 1 (level 2) if the checked variables of the engine torque, the speed difference between the shafts, and the feedback value are below the limit or have a value of 1. In the third stage, the driver presses the button and the algorithm changes the value *LockFI-II* to 1 (level 3) if the conditions are met as in the previous stage. These speed differences and drive torque limits are applied for safety reasons, so that an inexperienced driver does not lock the differential in a dangerous situation which might lead to the destruction of the powertrain. The feedback value represents the actual state of all-wheel drive activation or individual differential locking. If the user wants to activate all-wheel drive or the differential lock, the feedback value must first be changed to 1. If the driver does not need to activate all-wheel drive or lock the differential, the driver uses the button and switches to level 0. In general, the user should set the automatic control mode by default. Manual control mode should only be used by the user if this is unavoidable or if the system indicates a fault.

Generally, if the arrows in the diagram (Figure 3) are marked in red, the user moves up in the diagram. If the arrows are marked in blue, the user moves down in the diagram. This deactivates all-wheel drive and unlocks all the differentials. The black arrows mean movement up/down in the diagram. Green cells always show what should be locked or activated in the individual diagram position of the control algorithm.

The automatic control mode works according to right side of the diagram in Figure 3. The status evaluation is based on speed difference values, engine torque, feedback of all-wheel drive and differential locking, brake pedal values, clutch pedal values, accelerator pedal values, system errors, mode setting (road, field, terrain/snow), wheel and shaft speeds, pneumatic circuit pressure, the slip between wheels or shafts, the steering angle of the front wheels, time, and the test time. Of course, the variables always have some limits or values for control and decision. These values are not being published because they are confidential.

The diagram of the automatic control mode is divided into 9 levels corresponding to the activation of the *Lock FA*—all-wheel drive, the lock of the respective differentials *Lock R*—*Lock F*, or intervals of waiting—Slip Up/Down. 

General movement through the diagram is dependent on the setting of the control modes (road, field, terrain/snow). If the road mode is set, the diagram position of the control algorithm moves up to the maximum level of 2. If field mode is set, the diagram position of the control algorithm moves up to the maximum level of 5. The terrain/snow mode sets the control algorithm to move through the complete diagram.

In the first stage, all-wheel drive is deactivated and the individual differentials are unlocked (level 0).

If the vehicle wheels begin to slip under unfavorable adhesion conditions and the evaluated slip is above the specified limit, it is moved up to level 1 in the diagram of the control algorithm. At this stage, additional limits and conditions must be met: The accelerator pedal must be pressed, the speed difference must be less than the specified limit, the vehicle must not be braking, the vehicle velocity must be below the specified limits, the pneumatic pressure must be within the specified range, and the driving torque must be below the specified limit. It must not be braking in any way, which means that braking by a motor brake, a retarder, or a parking brake is also included in the braking variable *sigB*. The accelerator pedal value *sigT* must be 1 to move up to the higher level in the algorithm diagram. This means that if the driver presses the accelerator pedal, the input value *sigT* for the control algorithm is 1. Then, the algorithm knows that the potential slip of the wheels is justified. If the slip is evaluated and the accelerator pedal is 0, the algorithm cannot move to the next level. The vehicle velocity value *vc* determines whether the system can still be active to avoid locking the actuators at high vehicle speeds. The engine torque *Te* value is used for the protection of the powertrain against torque shocks. If everything is accomplished, the control algorithm moves up to Slip up FA (level 1). If any condition is not met, the position in the diagram moves down to level 0.

At level 1, the previous conditions and limits and the length of their duration is monitored. If this time is longer than the specified limit, the new condition is met and the diagram position moves up to level 2. The output signal *LockFAI-II* and *LockIR* is changed to 1 and sent to the actuator to activate all-wheel drive and to lock the rear inter-differential. 

At level 2, if any condition is not met, the position in the diagram moves down to level 3. If the conditions for moving the position of the diagram are met, it changes immediately (level 2). If the conditions are not met, the position of the diagram moves down after the specified time.

Another way to move up to level 2 regards the algorithm monitoring the tilt angle of the vehicle along its transverse axis using gyroscopes and accelerometers. This function has a higher priority than the previous conditions. If the angle value is greater than the specified parameters, the position of the diagram moves up to level 2. This function of monitoring the tilt angle was deactivated during the prototype testing. An advantage of this function is the uniform distribution of the drive torque on all wheels, which means that individual shafts are not overloaded. 

For a further move up to level 4 in the diagram, other conditions must be met. One is the limit for the steering angle of the wheels; if the steering angles *DeltaFLI* or *DeltaFRI* exceed the set limit, the algorithm does not enable a move up to level 4 to avoid damaging the powertrain. At level 4, the feedback value is also monitored. This means that the algorithm waits at the given position of the diagram until the individual feedback values *Feedback_i_* are changed to 1, which means that the differentials are locked or all-wheel drive is activated. Thus, the drive torque is appropriately distributed throughout the powertrain, protecting it from damage. If the conditions are met, it moves up to level 4 in the diagram. If the conditions are not met, it moves down in the algorithm to level 3 and then to level 0, according to the conditions and the limits of the levels. 

At level 4, the same conditions and limits as level 2 are monitored during the set timeout. If the conditions are met, it is moved up to level 5. If the conditions are not met, the position of the diagram moves down to level 2, and so on.

At level 5, the output signal *LockRI-II* is changed to 1 and sent to the actuator to lock the rear axle-differentials. In addition, the same conditions and limits are monitored with a different setting of control limit parameters. Therefore, according to the result of the evaluation of conditions and limits, the position in the diagram is moved down to level 6 or up to level 7. If unfavorable adhesion conditions persist, the diagram position moves up to level 8. This is the top level of the diagram.

At level 8, the output signal *LockFI-II* changes to 1 and is sent to the actuator to lock the front axle-differentials. All-wheel drive is activated and all the differentials are locked. At the same time, the algorithm does not know if wheel slip is still occurring. For this reason, the so-called test loop is programmed. If any conditions, errors, or limits do not specify that the diagram position moves down to level 5, it waits for the specified time interval and then it moves down to level 5. Subsequently, the slip is evaluated and if the slip continues, it moves up to level 8. If there is no slip, it continues to move down in the diagram. This assumes that the vehicle, as an example, has passed through muddy terrain and now the system reaction is no longer necessary. This movement in the diagram happens continuously according to actual adhesion conditions.

### 3.2. Error and Limits of the Control Algorithm

Error function is used for the evaluation of the possible failure of the mechatronic system. The control algorithm works according to the result. The basis is several vectors containing information about faults and system limits. This is divided into three types.

The highest priority is any type of fault in which the system is deactivated in both control modes (automatic and manual). These include a fatal system error, a failure of the user-configurable parameters, or a sub-function initialization error. In addition, the pressure in the pneumatic circuit is monitored. If the pressure value is below the set limit for a set length of time, a sensor or pneumatic circuit failure is assumed. If any of the above-mentioned faults occur, the drive system may be damaged. Therefore, in this state, the system is deactivated.

Faults of the second type include the disconnection of the actuator electrovalve, which is monitored by the function given by the electrical circuit. This might mean, for example, a cable break. An important fault which is monitored is the speed sensor. The steering wheel sensor is monitored by the sum of its two signals; the control code checks the sum of its signals and, if it is outside the specified range, a fault is detected. CAN messages are also monitored, where, if the CAN message with information on the driving modes of the touch display does not come back by the specified time, it is evaluated as a failure. This is also applicable to other CAN messages received from the engine and ABS ECU. Therefore, in this state, the system deactivates the automatic mode and remains operational in manual mode.

The final type of fault is where the system is not limited in both control modes. It is a fault detected in the activation/deactivation of all-wheel drive or in locking/unlocking of the differentials. This is monitored by parts of the actuator and the feedback switch.

The sum of these detected failures should cover the entire range of possible system failures. This prevents damage to the powertrain and reduces risk of injury. 

Because many drivers nowadays have insufficient experience, the driver is informed of any limitations of the mechatronic system. The limits are displayed on the touch display for the driver, including monitoring of the engine torque limitations. Other important limits include the monitoring of shaft speed differences and the steering angle of the front wheels. Therefore, there is a function to inform the driver of these limitations. The final monitored limit is vehicle velocity, meaning that if the vehicle drives above the specified velocity limit and a slip occurs, the system is deactivated and the driver is informed.

## 4. Prototype Testing

Prototyping was used to test the vehicle or other equipment without the need for an ECU prototype. This had considerable advantages in terms of saving development time and cost. Hardware for Hardware in the Loop—HIL testing was used as a replacement for the control unit where the developed control algorithm was implemented. This was already debugged by Model in the Loop—MIL testing (see [14,15]). A different vehicle and powertrain computational model was used (see [16,17]), and this could also be used to solve vibration (see [18,19]). The test loop consisted of a controller, vehicle electronics, actuators, sensors, and mechanical parts. The prototype of the vehicle incorporated all elements of the mechatronic system, except for the ECU prototype, which was substituted by hardware for HIL testing. Another advantage was that development could take place in parallel and, if this proved to be an inappropriate course of development, there was no need to go to the expense of developing the ECU prototype. Another reason for this was to test the electronic and mechanical elements of the system. It also allowed for further verification of the control algorithm of the developed mechatronic system. Most errors that occurred during development were discovered in MIL testing; a range of possible errors was also to be expected in prototyping, but on a smaller scale. Afterward, the system fault finding was likely to be negligible when testing a complete prototype of a vehicle.

### 4.1. Hardware for Prototyping

**Figure 4:**
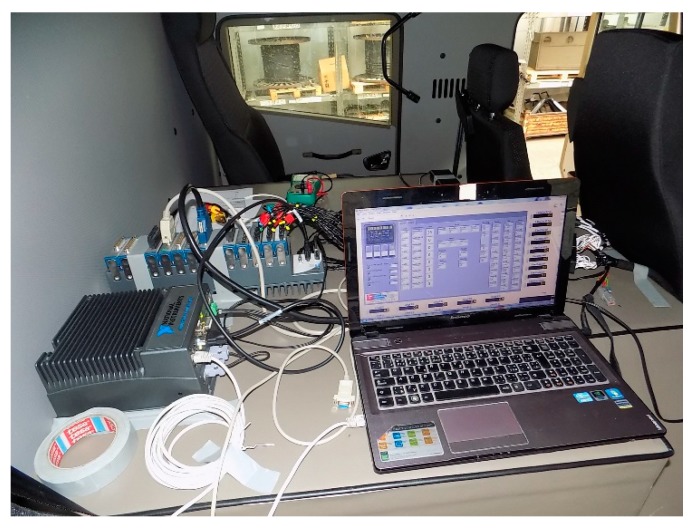
The hardware used for testing in the prototype vehicle.

Hardware from National Instruments was used for prototyping, see Figure 4. The basis was NI 3110RT hardware. A processor was located for the implementation of the developed control algorithm. The code was written in C/C++ language. A slot for module placement with inputs and outputs was connected to this main hardware, marked NI 9159. For this application, a slot with a field programmable gate array (FPGA) was used. 

In this FPGA, there was the programming of the communication of individual modules with the main hardware, which could be customized by the user. The FPGA was used to process fast processes. In the case of testing the developed system, the processing of wheel speed was programmed here. 

For the prototyping of the developed system, the following modules were used. Two NI 9229 modules were used for signal acquisition from the inductive wheel speed sensors, one NI 9239 module was used for sensing the analogue signal from a pneumatic pressure sensor, and two signals from the steering angle sensor. One NI 9425 module was used for pedal position sensing where values from CAN messages were not read and for values obtained from feedback switches in pneumatic cylinders. One NI 9472 module was used for switching system actuators. The last card used was the Peripheral Component Interconnect—PCI 8513/2, which was designed for CAN communication.

### 4.2. Software for Control Algorithm Implementation

The software used to communicate with the hardware was called NI VeriStand. There were various panels where the user set different specifics. This was the System Explorer interface. The compiled developed control algorithm was placed in the form of a model and its parameters were set. 

There was also an implementation of a FPGA program for communicating with the modules and code of processing signals from the wheel speed. The other settings were connected to the inputs and outputs of the control algorithm with the respective module channels and the wheel speed inputs. It was also important to create a database for CAN communication. Their specific signals were set and a conversion was carried out. A CAN communication interface was also set up. Then, at start-up, the model was placed on the hardware processor and the FPGA code was put into the FPGA slot. Subsequently, the NI VeriStand served as an imaging interface or an interface for setting the control algorithm parameters. There was also the possibility of placing a different control element for this system (manual and automatic controls). This was all created in the so-called Workspace.

### 4.3. Graphical Interface

**Figure 5:**
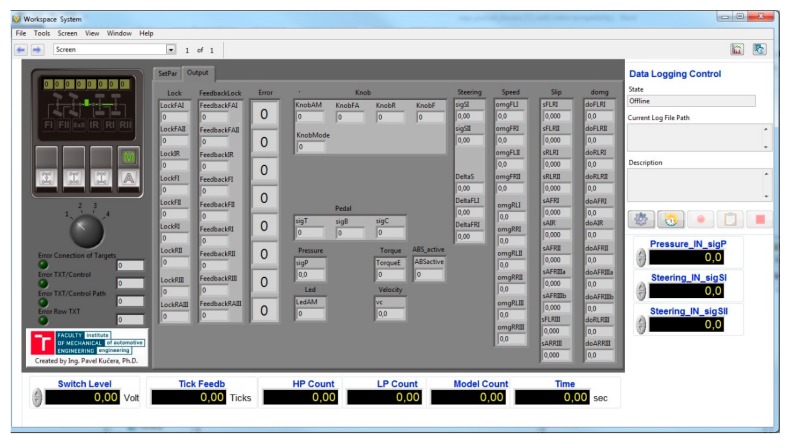
Workspace NI VeriStand software window with customized graphical elements for visualization and control of the algorithm.

The graphical interface for displaying actual values for testing was run through the so-called Workspace in NI VeriStand. The user operated pre-configured so-called Workspace Controls where graphs, value displays, value settings, buttons, etc. were defined. If the library with elements was inadequate, the user could design their own graphical elements. This was also the case for the development of the differential lock system. This interface was programmed in the NI LabVIEW software and is shown in Figure 5.

The control button and switches (automatic or manual control and manual actuator activation), the rotary switch (for the setting of control algorithm modes road, field and terrain/snow where the display is not used), the settings tab with control algorithm parameters, and the settings tabs with internal variables and output values sdre all available in the graphical interface. The Workspace window also used elements from its library to see if any loops in the hardware did not slow down. The last significant feature that was used was Data Logging Control for data monitoring during testing. The data were subsequently used to evaluate and check the proper functioning of the developed mechatronic system.

### 4.4. Real-Time Testing and Results

In terms of the tests, driving conditions on asphalt, field roads, and heavy terrain were tested. The tests included different ways of driving, starting, stopping, braking, turning, testing system failures, and testing on flat terrain and on special and inclined roads. In addition, the tests were conducted in different weather conditions, from high summer temperatures to rainy weather and snowy or icy roads. The testing also involved driving on a special wet road in a straight direction or turning. In this case, the maneuver was carried out on rugged terrain. The roadway was composed mainly of mud and small stones. Adverse weather in the form of mild rain was preferred. The shape of the testing road was a circuit with frequent changes of direction in driving and with different angles of tilt in the road. From the point of view of prototyping, the goal was to set up the control algorithm and test the mechanical, pneumatic, and electronic components of the developed mechatronic system. Therefore, various experiments using driving maneuvers were tested with wheel slip to monitor the correctness of the mechatronic system. The processing of the acquired data cannot be automated, therefore, the test sections where the system did not work optimally were selected. Then, the code was immediately modified and tested again. At the same time, the internal control parameters for the vehicle prototype were matched by these tests. Because the control algorithm and system operation were significantly debugged in the virtual environment by MIL testing, it was not necessary to make major changes to the system operation. Therefore, all functions were verified and sensor faults were simulated in the testing. The evaluation of data from one experiment is shown in the following figures.

**Figure 6:**
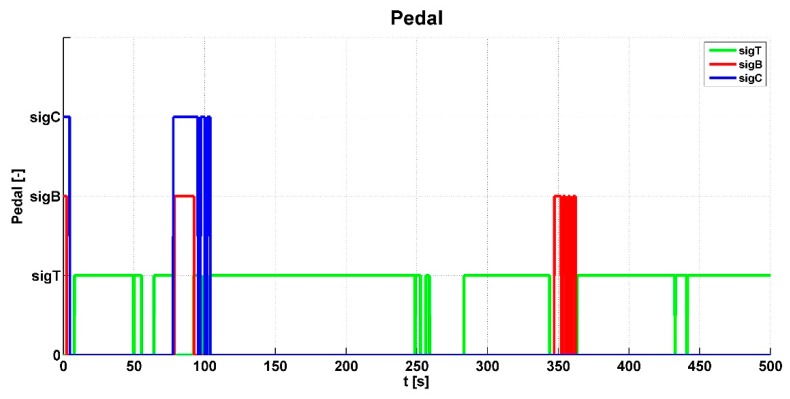
Pedal values.

**Figure 7:**
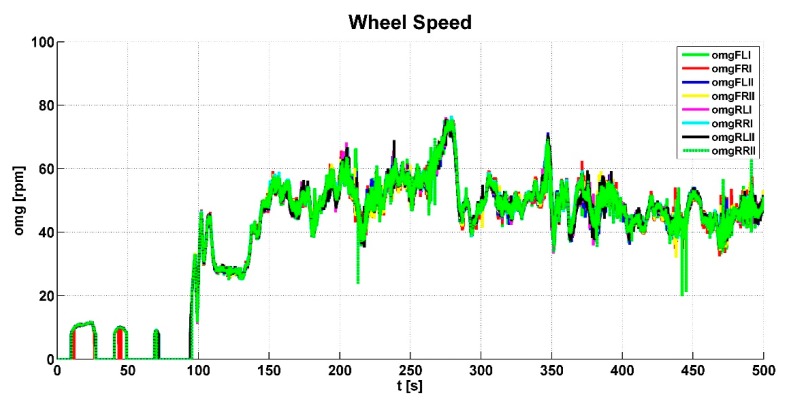
Wheel speed values.

**Figure 8:**
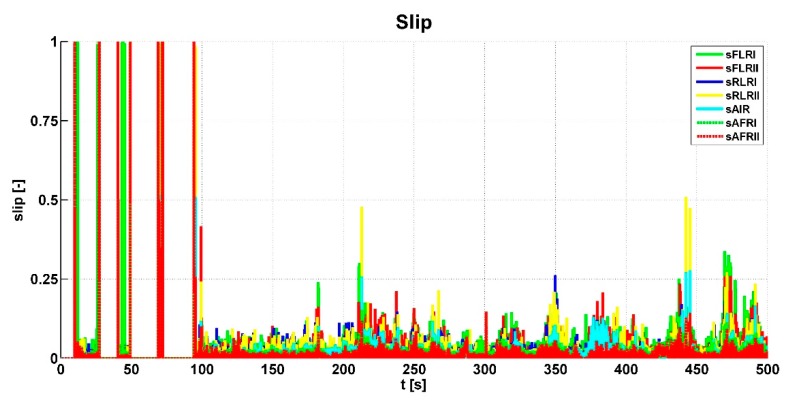
Slip values.

Figure 6 shows input signals for the control algorithm from the clutch pedal—*sigC*, the accelerator pedal—*sigT*, and the brake pedal—*sigB*. It can be seen in the graph that the vehicle prototype was started several times. At around 100 s, the gears were changed; braking was done at around 350 s. Figure 7 shows and evaluates the individual wheel speeds *omg_i_* that were inputs for the control algorithm. The algorithm evaluated the slip between the individual wheels and the shafts of the powertrain. This slip is shown in Figure 8.

**Figure 9:**
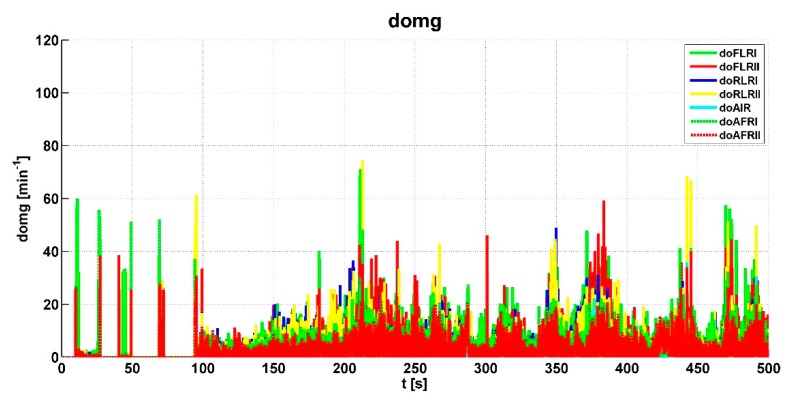
Values of shaft speed differences.

This slip is the main variable according to the response determined by the control algorithm. The graph shows higher peaks generated by the wheel slip, where the algorithm evaluated the output signal sent to the actuators. In addition, the values of shaft speed differences were evaluated from wheel speeds. The course of these variables can be seen in Figure 9.

**Figure 10:**
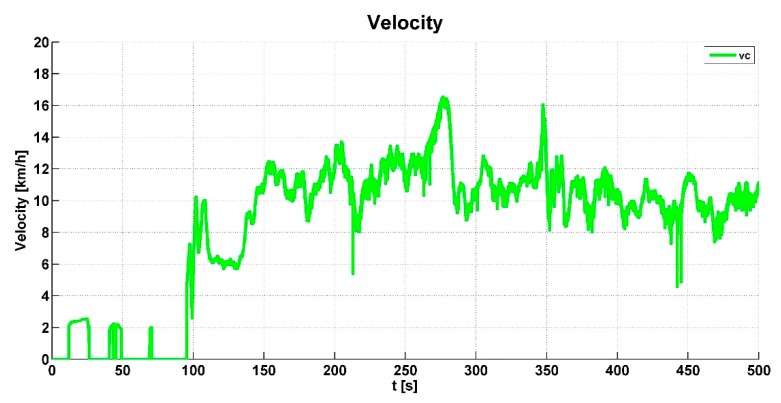
Vehicle speed value.

**Figure 11:**
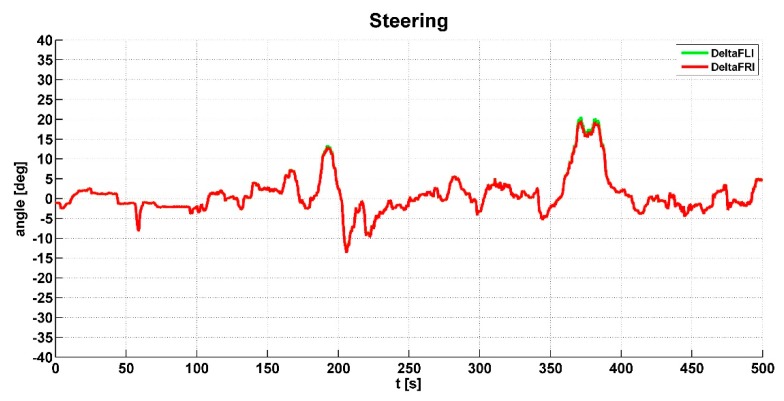
Values of the steering angle.

The vehicle speed *vc* shown in Figure 10 was also evaluated from the wheel speeds. Figure 11 shows the steering angle Delta of the right and left wheels of the front axle. This signal was used to calculate the slip correction and is included in Figure 8. This signal was also used to check the excess steering angle to lock the axle-differential. 

**Figure 12:**
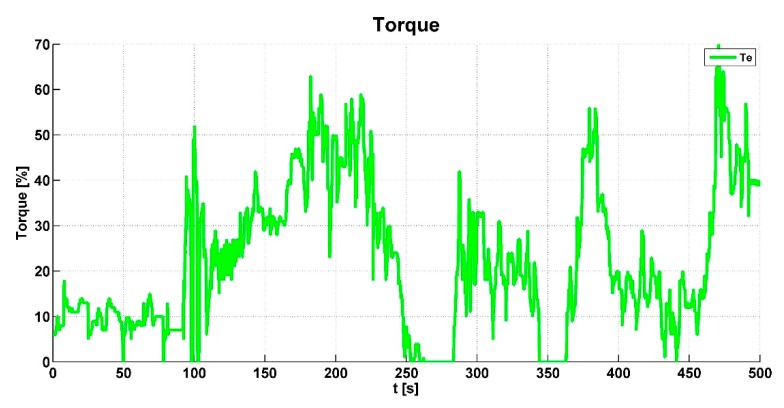
Engine torque value.

**Figure 13:**
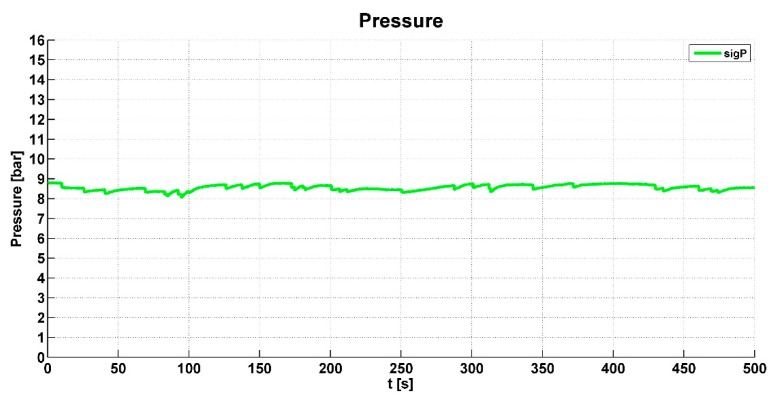
Air pressure value in the pneumatic circuit.

The output of the engine torque *Te* is shown in Figure 12. When the engine torque had a higher value than the specified limit, the algorithm responded accordingly. Figure 13 shows the pressure (*sigP*) in the pneumatic circuit. This graph presents whether the pneumatic system was sufficiently designed; the pressure was sufficient when all-wheel drive was intensively activated and the differential was locked.

**Figure 14:**
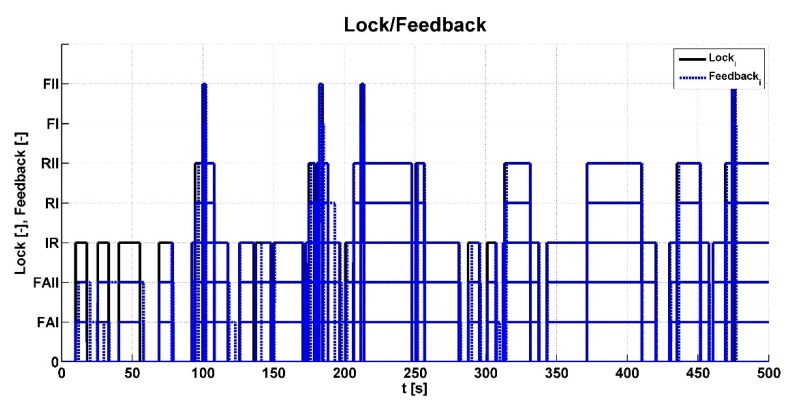
Status of locking/unlocking and feedback.

The main output data are the *Lock_i_* signals for actuator control (black curve) and *Feedback_i_* from the feedback switches sensor (blue curve). This is shown in Figure 14. A *Lock_i_* or *Feedback_i_* status with a value of 1 is represented by the indication of a particular actuator on the vertical axis. An *Unlock_i_* or *Feedback_i_* status with a value of 0 is on the vertical axis. It is clear from the graph that intensive interventions of the control system occurred in this case due to difficult muddy terrain.

In general, all data were analyzed and the correct control algorithm function was checked. In selected tests, manual monitoring of slipping and other variables occurred depending on whether the individual dog clutches needed to be locked. In other cases, a check was done depending on whether dog clutches did not need to be locked. If something was wrong, an adjustment was made.

**Figure 15:**
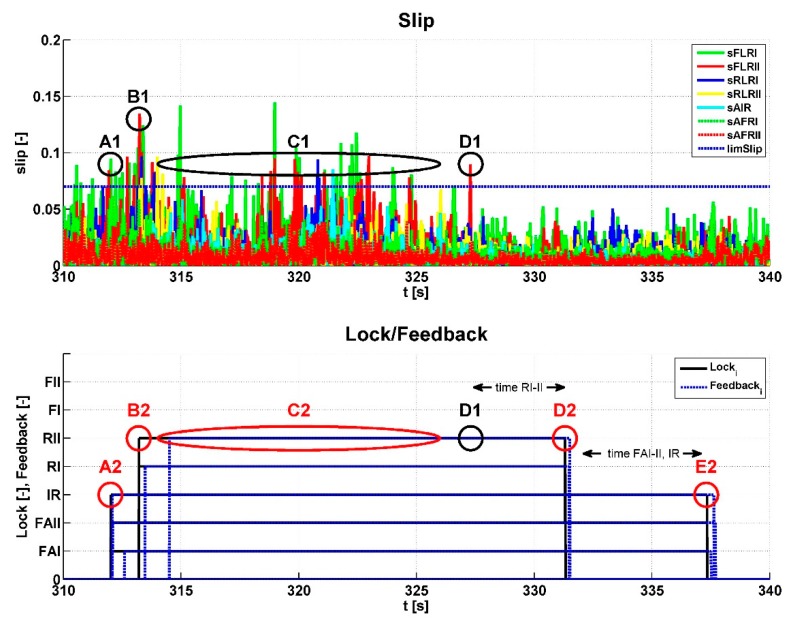
Status of lock (a value of 1 is represented by the indication of a particular actuator on the vertical axis)/unlock.

The procedure for checking the algorithm function is shown in Figure 15, with a time slice from the previous graphs. The *LockFAI-II* and *LockIR* signals were sent to the actuators in the lock graph at point A2. Therefore, the slip at point A1 was checked. This exceeded the specified slip limit, 0.07, for this test and the duration of this condition was longer than the set time limit. From this, we can conclude that the algorithm worked properly, assuming that other conditions were met. The *LockRI-II* signal was sent to the actuators in the Lock graph at point B2, therefore, the slip at point B1 was also checked. This exceeded the specified slip limit for this test. The duration of this condition was longer than the set time limit, therefore, the algorithm worked properly in this case as well. In addition, the slip was evaluated in the area of the graph marked C1, but the front axle differentials were not locked (C2). More detailed analysis revealed that the duration of all of the slip peaks was less than the set time limit. Therefore, the algorithm did not move up in the scheme of the algorithm and did not send a signal to the actuators. This situation was resolved correctly. At point D1, the last slip limit was exceeded, so the control algorithm moved down to level 6 and waited for a set time. Because another slip exceeding the limit did not occur, after four seconds (point D2), the control algorithm moved down to level 2 and level 3. At level 3, the same situation occurred and after six seconds (point E2), the control algorithm moved down to level 0. At all of the points mentioned, the control algorithm responded correctly. This algorithm function control was always applied in atypical situations. If an error was detected, the code was corrected and testing continued.

In the vehicle cab, the driver did not feel vibration and noise when locking the dog clutch. This was a positive result. One result of testing was also the debugging of the mechatronic system in terms of function and shock torque. Where the vehicle did not activate the system during testing, the vehicle got stuck on parts of the test road. The vehicle stopped gradually and remained stationary. From the graphs’ perspective, the results display a certain value of the speed of one wheel while the other wheel speeds would be zero. In the case of a vehicle without this system, the driver would have to activate all-wheel drive and lock the relevant differentials manually, as is the case in serial vehicles.

The main advantage of the developed system is the activation of all-wheel drive and the locking of individual differentials in the case of wheel slip under unfavorable adhesion conditions. It is a more efficient vehicle operation, which is economic and safe for the vehicle powertrain. Another important advantage is the recognition of the incline of the road, whereby the system appropriately distributes the torque in the powertrain without allowing wheel slip. A disadvantage of the system may be that the system does not work at a wheel-speed of zero due to the type of speed sensors, which are inductive sensors with output signals that have zero amplitude at speeds approaching zero. Therefore, it is advisable to consider using another type of sensor.

## 5. Conclusions

This article dealt with the development of a mechatronic system for locking differentials. It was designed for different types of vehicles, from trucks to special vehicles and tractors. The basic principle was wheel-speed monitoring, from which the slip was evaluated. Once the control algorithm evaluated the slip, it took the appropriate steps.

In the first phase of the development, a two-way control algorithm was proposed for a partially independent and a dependent automatic differential lock. This article described the so-called dependent control. The relevant section described its operating principle and the required inputs and outputs for actuators.

The main part of the article was devoted to the verification of the mechatronic system using prototyping. This involved the use of hardware for HIL testing, which served as a replacement for the ECU prototype. A vehicle prototype was available and this system was used with it. Experiments were conducted on different road surfaces and under severe weather conditions. The recorded test data were evaluated and adjustments to the mechatronic system were made. At this stage of testing, an estimated 28% of errors were detected. In particular, the testing of a mechatronic system similar to serial production was carried out. The main output was to debug the mechatronic system according to the operating requirements.

In summary, this work described a mechatronic system that was developed for locking differentials, including the testing of it on a vehicle prototype. The experiments and the customers’ responses show that the system worked well and was beneficial to vehicles, preventing the vehicles from jamming in conditions of unfavorable terrain. At the same time, it protected the powertrain against differential locking by inexperienced drivers.

Development will continue with the use of the system in other types of vehicles and cooperation with other customers. We predict the next development to occur will be the use of so-called neural networks to allow the control algorithm to learn and adapt to vehicle and driving situations during its use.
